# Perineuriomatous Melanocytic Nevus: A Case Report of a Rare and Underreported Melanocytic Lesion

**DOI:** 10.3390/dermatopathology13020025

**Published:** 2026-05-30

**Authors:** Muhammad N. Mahmood, Eunice Y. Chow

**Affiliations:** 1 Department of Laboratory Medicine and Pathology, University of Alberta Hospital, Edmonton, AB T6G 2B7, Canada; 2 Division of Dermatology, Department of Medicine, University of Alberta, Edmonton, AB T6G 2B7, Canada; eunicew@ualberta.ca

**Keywords:** perineuriomatous melanocytic nevus, neurotized nevus, perineurioma, desmoplastic melanoma, BRAF, dermoscopy

## Abstract

Melanocytic nevi exhibiting perineuriomatous differentiation are rare and pose significant diagnostic challenges due to their low incidence and morphological resemblance to other cutaneous spindle-cell lesions, including desmoplastic melanoma. In this report, we describe the clinical, dermoscopic, microscopic, and immunohistochemical features of a perineuriomatous melanocytic nevus on the right mid-forearm of a 73-year-old Caucasian man. Given the scarcity of reported cases, documenting additional examples is crucial for refining clinicopathological diagnostic criteria. Accurate identification relies on thorough histopathological and immunohistochemical assessment. By presenting the current case, we aim to enhance diagnostic accuracy and raise awareness of this uncommon nevus. A clearer understanding of these characteristics will help dermatologists and dermatopathologists identify this nevus and distinguish it from other cutaneous spindle-cell proliferations.

## Simple Summary

Perineuriomatous melanocytic nevus is a rare, harmless skin growth first described in 2011. It shows traits of both perineurioma and melanocytic nevus. As these traits are uncommon and easy to miss, this growth is often mistaken for other spindle-cell skin growths like blue nevus, dermatofibroma, neurofibroma, or desmoplastic melanoma. Due to its rarity and resemblance to other skin growths, perineuriomatous melanocytic nevus is often misdiagnosed. In this report, we present a case of a right forearm lesion of this growth in a 73-year-old man and share clinical and laboratory findings to help doctors identify and distinguish it from similar skin lesions.

## 1. Introduction

Perineuriomatous melanocytic nevus (PN), also referred to as melanocytic nevus with perineuriomatous differentiation, is a rare benign melanocytic lesion characterized by prominent perineurial differentiation [1]. Spindle-cell differentiation often occurs in both congenital and acquired nevi, occasionally representing neurotization with Schwannian or Meissnerian differentiation of melanocytes [2,3]. In contrast, neurotization with perineuriomatous differentiation of melanocytes is uncommon and remains insufficiently documented in the literature.

First described in 2011, PN is characterized by a combination of perineurioma and melanocytic nevus components [1]. It displays a biphasic microscopic pattern with melanocytic nests and, typically deeper, spindle cells arranged in a whorled pattern in a myxocollagenous stroma. Perineuriomatous differentiation in melanocytic nevi is likely underdiagnosed, as it can be mistaken for other cutaneous spindle-cell proliferations. The perineurial component may resemble other cutaneous stromal or neural proliferations, leading to confusion with blue nevus, dermatofibroma, neurofibroma, sclerosing nevus, desmoplastic melanoma (DM), or other dermal stromal proliferations [4]. A possible relationship between neurocristic cutaneous hamartoma and PN has also been suggested [5].

Awareness and overall understanding of this proliferation remain limited due to the small number of cases reported in the literature. Herein, we present a case of PN on the right mid-forearm of a 73-year-old Caucasian man, emphasizing its clinical presentation, histopathological features, and immunohistochemical profile. Greater clarity regarding these characteristics will assist dermatologists and dermatopathologists in identifying and distinguishing PN from other cutaneous spindle-cell proliferations.

## 2. Detailed Case Description

A 73-year-old man presented to the dermatology clinic for follow-up evaluation of previously diagnosed melanomas. His medical history was notable for melanoma of the right retroauricular scalp (3 mm Breslow thickness) diagnosed in 2022, which was managed with wide local excision, sentinel lymph node biopsy, and adjuvant nivolumab therapy. The sentinel lymph node was negative for metastasis. In 2025, a second melanoma was identified on the right shoulder (0.3 mm Breslow thickness) and was treated with wide local excision. Additional diagnoses included prostatic adenocarcinoma (2019), adenocarcinoma of the lower esophagus (2024), and brain metastasis to the right frontal lobe (2025) of unknown origin, presumed to be metastasis of melanoma or esophageal adenocarcinoma, treated with gamma-knife. The patient also had a history of benign nevi (including two biopsy-proven blue nevi on the right temple and left dorsal foot) and multiple basal cell carcinomas. He denied constitutional symptoms such as unintentional weight loss, night sweats, or unexplained fevers.

### 2.1. Clinical Examination

**Figure 1:**
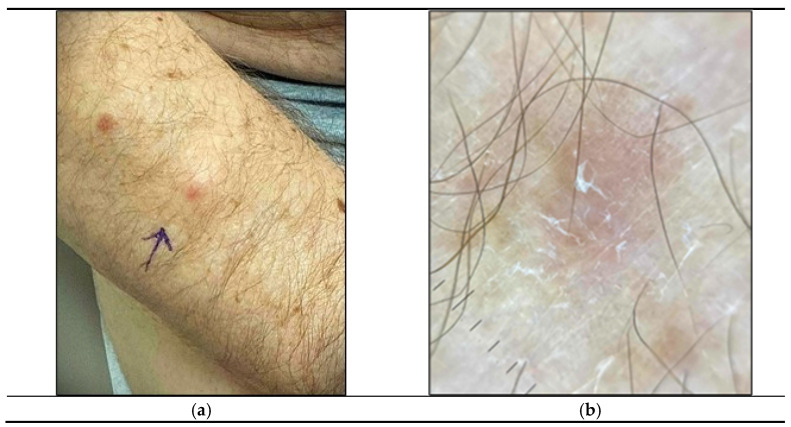
Clinical appearance of PN on the right mid-forearm: (**a**) A non-tender 7 mm erythematous pink dermal papule (without epidermal changes), overlying a poorly demarcated skin colored subcutaneous nodule; (**b**) Dermoscopy revealed white scale secondary to background xerosis, and an ill-defined faint light-brown patchy peripheral pigment network (more defined along the superior edge and less defined inferiorly), with a central amorphous pink background.

The patient was alert and oriented, with no signs of distress. There was no clinical evidence of melanoma recurrence, satellite lesions, or in-transit metastases around the previous melanoma excision sites. Lymphadenopathy was not present. Multiple seborrheic keratoses and cherry angiomas were observed on the torso. Two pigmented papules, consistent with nevi, were identified on the lower back. On the extensor surface of the right mid-forearm, a non-tender, 7 mm erythematous pink dermal papule (without epidermal changes), overlying a skin colored, poorly demarcated subcutaneous nodule, was detected (Figure 1a). Dermoscopy revealed white scale secondary to background xerosis and an ill-defined, faint, light-brown, patchy peripheral pigment network with a central amorphous pink background (Figure 1b). It did not exhibit a “dimple sign.” The patient did not know how long it had been present, but it was not noted at his previous visit 6 months prior. The clinical differential diagnosis for this lesion included cyst, lipoma, and cutaneous metastasis from prior melanomas or adenocarcinomas. An 8 mm punch biopsy of the forearm lesion was performed, and the pigmented papules on the lower back were also biopsied.

### 2.2. Histopathologic Findings

**Figure 2:**
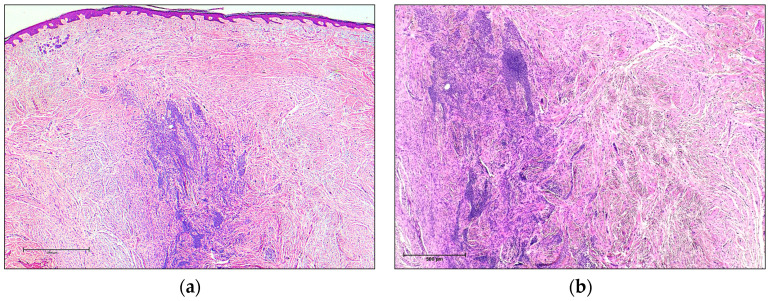

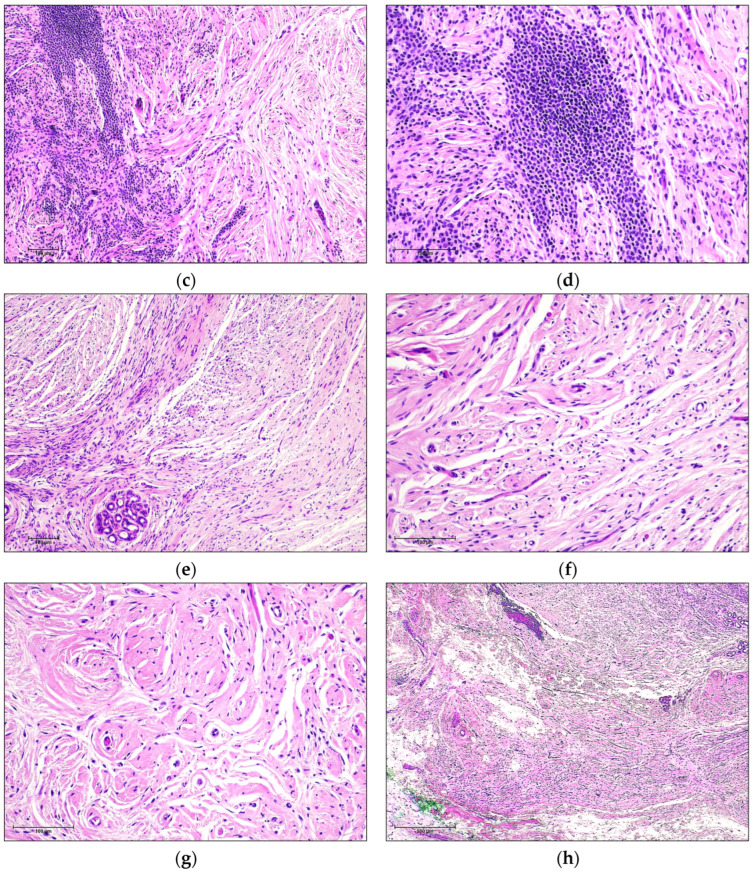
Microscopic features of PN: (**a**) Amelanotic biphasic dermal proliferation (H&E, ×20); (**b**,**c**) Biphasic population of cells showing transition between banal nevoid melanocytes (left side of image) and bland uniform spindle cells (right side of image) (H&E, ×40 and ×100, respectively); (**d**) Centrally, round-to-ovoid nevoid melanocytes without cytological atypia or mitotic activity (H&E, ×200); (**e**,**f**) Spindle-cell component formed by bland, uniform slender stellate cells arranged in short fascicles within a fibrotic stroma (H&E, ×100 and ×200, respectively); (**g**) Whorled arrangement of spindle cells with wavy nuclei observed in a densely hyalinized stroma (H&E, ×200); (**h**) The spindle-cell perineuriomatous component extend into subcuteous fat (H&E, ×40).

Histological examination revealed an amelanotic dermal proliferation extending into the subcutis (Figure 2a–c,h). The tumour thickness measured 6 mm, and the lesion displayed a biphasic morphological pattern. Centrally, bland, round-to-ovoid nevoid melanocytes were present without cytological atypia or mitotic activity (Figure 2d). At the periphery and within the subcutis, the lesion was formed by slender, stellate spindle cells (Figure 2e,f). A transition from the nevoid component to a nodular spindle-cell proliferation was evident in the peripheral and deeper regions (Figure 2b,c). The spindle cells were bland and uniform, arranged singly and in short fascicles within a loose fibromyxoid stroma. In certain areas, a whorled arrangement of spindle cells with wavy nuclei was observed in a hyalinized stroma (Figure 2g). Peripheral lymphoid aggregates were absent. Hypercellularity and increased mitotic activity were not identified within the spindle-cell component. An intraepidermal component was not identified.

### 2.3. Immunohistochemical Findings

**Figure 3:**
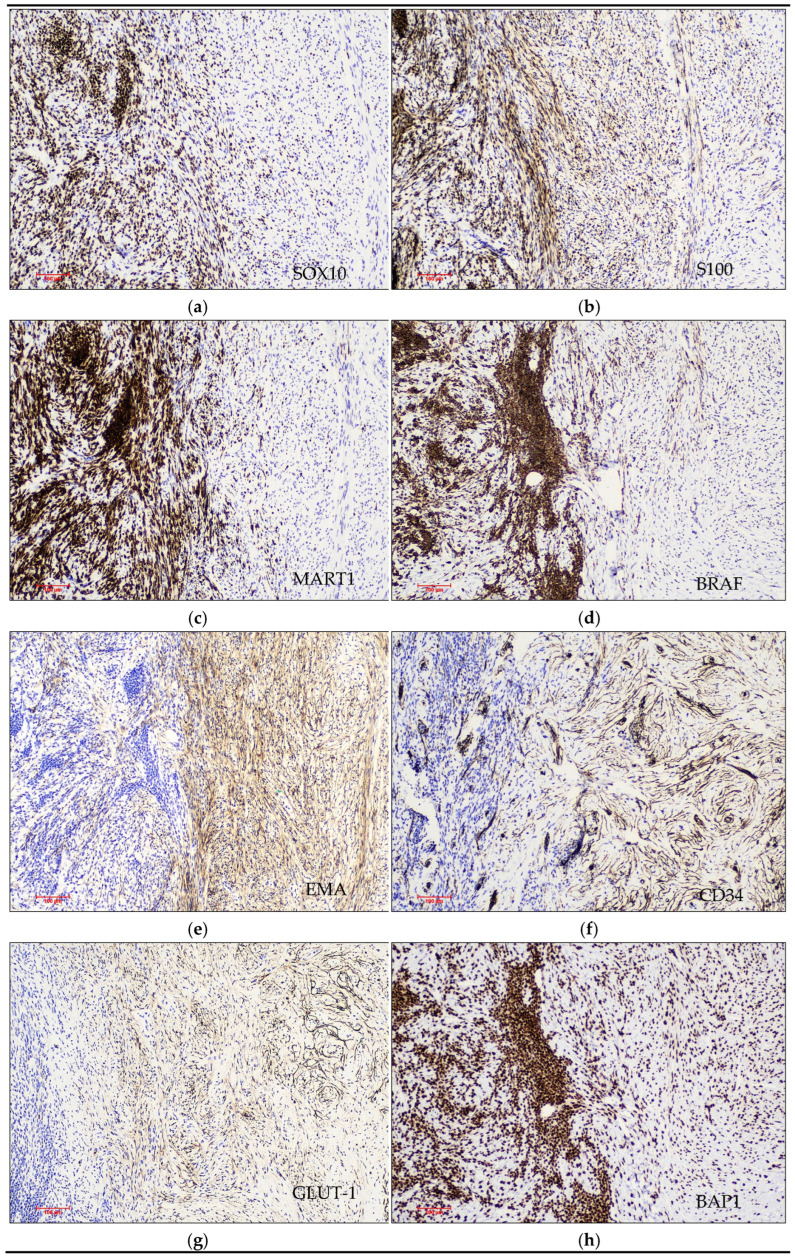
Immunohistochemical profile of PN: (**a**–**d**) SOX10, S100, MART1, and BRAF V600E stains showed a similar pattern of staining, with the stains being strongly positive in the conventional nevus component (left half of images) and loss of staining noted in the perineuriomatous spindle-cell component (right half of images) (×100); (**e–g**) EMA, CD34 and GLUT-1 stains showed a similar pattern of staining, with positive staining being noted in the perineuriomatous spindle-cell component (right half of images) and loss of staining noted in the conventional nevus component (left half of images) (×100); (**h**) BAP1 stain showed positive staining in both components of the nevus (×100).

Immunohistochemical analysis revealed strong positivity for SOX10, S100, and MART-1 in the conventional nevus component (Figure 3a–c). Occasional scattered spindle cells also showed positivity for SOX10, S100, and MART-1, although the majority of spindle cells were negative. EMA highlighted delicate, elongated cytoplasmic processes arranged in a reticular network within the spindle-cell component (Figure 3e). Both GLUT-1 and CD34 were positive in the spindle-cell component, with CD34 exhibiting a fingerprint staining pattern (Figure 3f,g). EMA, GLUT-1, and CD34 were negative in the nevoid component. HMB45, PRAME, and desmin were negative in both components. p16 and BAP1 showed retention in both nevoid and spindle-cell components (Figure 3h). BRAF V600E immunostain was strongly positive in the nevoid component, with only occasional positive cells in the perineuriomatous component (Figure 3d). Ki67 indicated a very low proliferative index (<1%) in both components. Claudin-1 staining was not performed because it was not available at our institution.

### 2.4. Pathological Diagnosis

The lesion on the right mid-forearm was diagnosed as a PN. The two lesions on the lower back were diagnosed as banal compound melanocytic nevi. Features associated with metastatic melanoma were not seen. These findings did not necessitate further specific management. The patient was advised to attend regular melanoma surveillance visits.

## 3. Discussion

The current literature on PN consists of a limited number of case reports and case series [1,4,6,7,8,9,10]. This limited documentation implies that PN is either exceptionally rare or frequently underdiagnosed. The current literature describes different aspects of PN as follows:

### 3.1. Clinical Presentation

The majority of PN cases are identified in referral settings rather than during routine pathology sign-outs. These lesions predominantly affect middle-aged adults of both sexes and demonstrate a broad anatomical distribution [4,6,7]. They are more frequently observed in sites with intermittent sunlight exposure. McAfee et al. report a median age at diagnosis of 42.5 years (range 25–64) in a series of eight cases, with an equal distribution between sexes [6]. Lesions are identified on the arm (*n* = 4), trunk (*n* = 2), and head and neck (*n* = 2), with a median size of 7.5 mm (range 5–12 mm).

Most reported PN cases are solitary. However, one report describes a patient with multiple soft, pale papules on the trunk resulting from a germline mutation [8]. Follow-up data are limited, but no adverse outcomes have been documented. In Ferreira et al.’s case series, all patients were alive and disease-free at follow-up (median: 13 months; mean: 18 months) [4]. Four lesions were completely removed at biopsy, and two were partially sampled, with no recurrence or metastasis. One patient treated for presumed DM had a negative axillary sentinel lymph node biopsy.

### 3.2. Histopathologic Profile

All cases of PN exhibit a biphasic histomorphological profile [4,6,7]. The nevus is primarily situated in the dermis, with occasional extension into the upper subcutis. The superficial or central component consists of dermal nevoid melanocytes, while the deep or peripheral component comprises spindle cells arranged in fascicles or whorls. Nevoid melanocytes in the reticular dermis gradually merge with the spindle-cell population. The spindle cells are cytologically bland and typically organized in short fascicles within a collagenous or fibromyxoid stroma. Lymphoid aggregates can be present; however, cytological atypia, pleomorphism, and increased mitotic activity are generally absent [4]. Almashad et al. found that three of four cases have a smaller junctional component than the dermal component, a configuration termed the “reverse shoulder” phenomenon by the authors [7].

### 3.3. Immunohistochemical Profile

The melanocytic markers S100, SOX10, MART-1, and HMB45 stain the conventional nevus component, but they are negative or only focally positive in the spindle-cell component, indicating a loss of melanocytic differentiation within the perineuriomatous area [4,6,7]. In contrast, the perineuriomatous spindle-cell component is positive for EMA, CD34, Claudin-1 and GLUT1, with CD34 staining in a fingerprint-like pattern. Additionally, the Ki67 proliferation index remains low (<5%), while p16 expression is retained in both components, and p53 is negative. Most cases exhibit the BRAF V600E mutation in conventional nevus cells, though variable or loss of expression is observed in the spindle-cell component, suggesting the divergence or downregulation of this mutation in perineural areas.

### 3.4. Differential Diagnosis

The histopathological differential diagnosis of PN among benign lesions includes conventional neurotized nevus, blue nevus, sclerosing or desmoplastic nevus, neurofibroma, and perineurioma. The spindle-cell component of PN may show focal SOX10 and S100 immunoreactivity; however, most perineuriomatous spindled cells do not express melanocytic markers. In contrast, the spindled components of other nevi in the differential diagnosis usually retain expression of melanocytic markers [11].

The differential diagnosis among malignant entities mainly includes DM. In Ferreira et al.’s series of PN, in three of five cases, the initial submitting diagnosis of referred cases was DM [4]. However, DM can be distinguished from PN by its demographic features, cytological atypia, and immunohistochemical findings. Pure DM usually occurs in older patients on sun-damaged skin of the head and neck [12,13]. It shows cytologically atypical spindle cells with mitoses and may have an overlying melanoma in situ. The spindle-cell component of DM is strongly positive for S100 and SOX10, but generally negative for MART-1, HMB45, and BRAF V600E. By contrast, the spindle cells in PN lose staining for S100 and SOX10. An increased Ki67 index in DM can also help differentiate it from PN. Peripheral lymphoid aggregates are unreliable for distinguishing between PN and DM, as they may occur in both conditions. Likewise, EMA expression is seen in both PN and DM, and is not specific to PN [14].

In our case, the patient’s history of melanomas raises a primary clinical concern regarding the potential for metastatic melanoma. However, several microscopic and immunohistochemical features argue against this diagnosis. Specifically, bland cytology, absence of mitoses, and a biphasic transition pattern do not support metastatic melanoma. Additionally, a very low Ki67 proliferative index and retained p16 expression further support the benign nature of this lesion.

The clinical, microscopic, and immunohistochemical findings in our case are consistent with those reported in previous studies of PN. Clinically, the lesion exhibits features of a stromal dermal and subcutaneous proliferation rather than those of a characteristic pigmented melanocytic lesion, with a discrete 7 mm pink papule overlying a larger, ill-defined subcutaneous nodule. This configuration is reflected histologically by the amelanotic dermal proliferation extending into the subcutis, where the deeper spindle-cell component accounts for the palpable subcutaneous nodule, and the more superficial nevoid component for the overlying pink papule.

Dermoscopically, the lesion shows a faint, patchy peripheral pigment network; a central amorphous pink background; and a non-specific vascular pattern. While a peripheral pigment network can be seen at the edge of a dermatofibroma, the absence of a central white, scar-like patch on dermoscopy, together with the absence of a “dimple sign” on lateral compression clinically, argues against this diagnosis. The patchy peripheral network corresponds to focal background basal pigmentation at the lesional edge rather than a true lesional junctional component, consistent with the absence of junctional nests on histology. The central amorphous pink background reflects the amelanotic dermal component visualized through an essentially uninvolved epidermis. The deeper subcutaneous spindle-cell proliferation lies beyond the depth of dermoscopic visualization, even with polarized optics, and accounts for the palpable, ill-defined subcutaneous nodularity appreciated clinically. Notably, the visible 7 mm papular footprint substantially underrepresents the true lesional size, which is more accurately appreciated by palpation than by surface inspection or dermoscopy. To the best of our knowledge, this represents the first dermoscopic description of PN in the literature, and the findings reflect its predominantly amelanotic, stromal nature rather than a conventional pigmented melanocytic lesion.

## 4. Conclusions

PN is a rare and distinct variant of benign acquired neurotized melanocytic nevus characterized by a specific histopathological and immunohistochemical profile (Table 1). This report details the clinicopathological features of a new case, thereby contributing to the existing literature on PN. Accurate diagnosis depends on thorough histopathological assessment and targeted immunohistochemical analysis. Increased awareness is particularly crucial for lesions that are partially sampled, exhibit a deep spindled component, or contain lymphoid aggregates that may mimic DM. The proper identification of PN is essential to prevent misdiagnosis and avoid unnecessary aggressive treatment.

## Figures and Tables

**Table 1:** Summary of the clinical, histopathological, and immunohistochemical features of PN.

**Clinical presentation**	- Usually longstanding, non-tender, papulonodular, dome-shaped, deep extension, appears dermal and subcutaneous - Median age between 42.5 years and 64.5 years, likely equal sex distribution, rare occurrence - Site: More commonly on sites with intermittent sunlight exposure (cases reported on arm, trunk, and head & neck) - Usually solitary, one case with multiple lesions reported
**Synonym**	- Melanocytic nevus with perineuriomatous differentiation
**Size**	- Median size: 7.5 mm (range 5–12 mm)
**Histopathology**	- Biphasic histomorphology, primarily situated in the dermis with occasional extension into the upper subcutis - **Conventional nevus component:** Superficial or central, round-to-ovoid nevoid melanocytes, merge with the perineuriomatous component - **Perineuriomatous spindle-cell component:** Peripheral or deep, slender stellate spindle cells, arranged in short fascicles or whorls, with collagenous or fibromyxoid stroma - Lymphoid aggregates can be present - No cytological atypia, pleomorphism, or increased mitosis - Junctional melanocytic proliferation can be seen, usually focal and lentiginous
**Histopathological differential diagnosis**	- Benign: Conventional neurotized nevus, blue nevus, sclerosing or desmoplastic nevus, neurofibroma, perineurioma - Malignant: Desmoplastic melanoma, metastatic melanoma
**Immunohistochemistry**	- **Conventional nevus component:** S100+, SOX10+, MART-1+, HMB45+/−, BRAF V600E+ - **Perineuriomatous spindle-cell component:** EMA+, CD34+, Claudin-1+, GLUT-1+, only rare cells positive for S100, SOX10, MART1, BRAF V600E - Both components are positive for p16 and BAP1 - Both components are negative for PRAME and p53
**Clinical behavior**	- Benign, no adverse outcomes reported

## Data Availability

All the data generated from this study are reported in the manuscript.

## Abbreviations

The following abbreviations are used in this manuscript:

PN	Perineuriomatous melanocytic nevus
DM	Desmoplastic melanoma
